# Targeting blood tests in the ICU may lead to a significant cost reduction

**DOI:** 10.1186/cc13205

**Published:** 2014-03-17

**Authors:** R Gray, F Baldwin

**Affiliations:** 1 BSUH, Brighton, UK

## Introduction

Daily blood tests are an essential diagnostic tool and routine practice in the management of ICU patients, but are associated with cost and a risk of anaemia. There is no evidence for the frequency or breadth of blood testing but there is evidence that by rationalising blood tests, significant cost saving can be made without a negative effect on mortality or length of ICU stay [1]. Blood tests in our ICU are requested by nurses, this is a unique practice to the ICU compared with other hospital departments.

## Methods

We had previously carried out a survey to assess blood requesting practise in UK ICUs. This demonstrated that routine tests were, as in our ICU, nurse led and the majority of staff underestimated the costs of tests. Using the results of this survey and a literature review we developed routine blood test guidelines for our general mixed 26- bed ICU/HDU. Following a period of staff education, we audited our blood requesting practice over 28 days.

## Results

Our blood testing did not comply with our guidelines. Over the 28-day period, €12,849.96 was spent on all blood tests. According to our guidelines, €2,914.96 was spent on inappropriate tests (€37,998.63 per year) (Table 1). Over 40% of this cost was spent on the coagulation screen.

**Table 1:** 

Test	Number of Test inappropriate requests	28-day cost (€)	Annual cost (€)
Full blood count	4	21.82	284.44
Urea and electrolytes	6	29.44	383.77
Liver function tests	95	583.18	7602.1
Coagulation screen	117	1276.86	16644.78
C-reactive proteir	45	245.55	3200.91
Bone profile	112	274.75	3581.56
Magnesium	88	483.35	6300.81

## Conclusion

In our ICU, unnecessary blood tests have a significant cost burden of approximately €38,000 per year. To limit unnecessary costs, consultants now lead requesting by completing a blood test *pro forma *on the evening ICU ward round indicating which blood tests are clinically needed the following day.
